# Review of Twin Pregnancies in a Tertiary Hospital in Abuja, Nigeria

**DOI:** 10.3329/jhpn.v31i2.16392

**Published:** 2013-06

**Authors:** Godwin Otuodichinma Akaba, Teddy Eyaofun Agida, Olatunde Onafowokan, Richard A. Offiong, Nathaniel D. Adewole

**Affiliations:** Department of Obstetrics and Gynaecology, University of Abuja Teaching Hospital, Gwagwalada, Abuja, Nigeria

**Keywords:** Delivery, obstetric, Pregnancy, twin, Nigeria

## Abstract

Studies on twin pregnancy are uniquely important to Africa and particularly Nigeria where the highest incidence in the world exists. This study was designed to determine the trend, rate, and obstetric outcomes of twin deliveries in the University of Abuja Teaching Hospital, Gwagwalada. This was a retrospective study of twin deliveries in the hospital over a period of 10 years. During the study period, there were 349 twin births out of 10,739 deliveries, giving an overall twining rate of 32.5 per 1,000 deliveries. Preterm delivery occurred in 39.7% cases and was, therefore, the most common complication. Mode of delivery was vaginal in 72.7% while 27.3% were delivered by caesarean section. Emergency caesarean section for delivery of both the babies was carried out in 22.3% while elective caesarean section for both the babies accounted for 1.0 %. Combined vaginal and abdominal delivery occurred in 4.0% of deliveries. The stillbirth rate was 102 per 1,000 births. There were 24 (8.0%) and 37 (12.3%) stillbirths among the first and the second baby respectively. The mean foetal weight was 2.395±0.63 kg while the female-to-male ratio was 1:1.1. The rate of twin deliveries in our centre is high. Successful vaginal delivery of twins is high when the mothers are booked and the presentations of the twins are favourable. The use of antenatal care services and good intrapartum management will help improve outcome in twin pregnancies.

## INTRODUCTION

Twins have been the object of great interest and fascination as well as intensive enquiry since ancient times (1). Twin pregnancy is associated with increased maternal and perinatal morbidity and mortality as well as healthcare costs (1,2,3).

Women with multiple gestations are nearly six times more likely to be hospitalized due to complications during pregnancy (2); ^  ^perinatal mortality rates are four times higher in twin babies than in singletons (3).

The incidence of twining is the greatest among blacks, least common in Asians, and of intermediate occurrence in whites (4). The incidence is 1.3 in 1,000 births in Japan,12 in 1,000 births in the United States, and the highest in Africa where an incidence of up to 49-53 per 1,000 births have been reported among the Yoruba's in South-West Nigeria (4).

Studies on twin pregnancy are uniquely important to Africa and particularly Nigeria where the highest incidence in the world exists. This study was designed to determine the trends, rate, and obstetric outcomes of twin deliveries in the University of Abuja Teaching Hospital, Gwagwalada.

## MATERIALS AND METHODS

The study was a retrospective review of all twin deliveries at the University of Abuja Teaching Hospital over a period of 10 years between 1 January 1998 and 31 December 2007. Data were retrieved from patient's case-notes and supplemented by information from the labour ward, postnatal ward, theatre, and medical record department. Only 300 case-notes had adequate information and were used for analysis in this study.

The collected data were entered into a computer, and statistical analysis was done using SPSS for Windows (version 15). Statistical significance was set at the p value <0.05.

## RESULTS

During the period under review, there were 349 twin births in 10,739 deliveries, giving an overall twining rate of 32.5 per 1,000 deliveries. The yearly frequency of twin deliveries during the 10-year period is shown in Table 1 and the figure. It can be inferred that there was an increasing trend in the last three years of the study, although the rate of twin deliveries generally was fluctuating. The age distribution and parity of the mothers are shown in Table 2. Their age ranged from 15 to 42 years. The mean age of the mothers was 28.4 years (±SD 4.69). The incidence peaked among the 25-29 years group which constituted 39%. Majority of the mothers—165 (55.0%)—were booked while 135 (45.0%) were unbooked. The mean parity of the women was 2.0±1.84).

**Table 1.** Yearly frequency of twin deliveries

Year	Total number of deliveries	Total number of twin births	Percentage
1998	874	26	2.97
1999	714	31	4.34
2000	814	17	2.09
2001	1,089	33	3.03
2002	764	33	4.31
2003	1,091	29	2.66
2004	1,211	49	4.05
2005	1,263	34	2.69
2006	1,447	43	2.97
2007	1,472	54	3.67
Total	10,739	349	3.25

Preterm delivery was the commonest complication occurring in 39.7% of the cases. This was followed by hypertensive disorders in pregnancy (pregnancy-induced hypertension, pre-eclampsia, and eclampsia), malpositions, and prolonged labour occurring in 9.3%, 8.0%, and 4.0% respectively. Cord prolapse complicated 3.0% of the cases. There were no complications in 27.6%. This is shown in Table 3.

Cephalic presentation of both the babies occurred in 47.0% of the cases while breech-breech presentation was seen in 9.3%. Cephalic-breech presentation was seen in 19.3% while breech-cephalic presentation occurred in 18.7% of twin deliveries. Other presentations (cephalic-transverse, breech-transverse, transverse-breech, transverse-cephalic) accounted for the remaining 5.7%.

Overall, spontaneous vaginal delivery was the mode of delivery in 170 (56.7%) and 148 (49.3%) for the first and the second baby respectively while assisted breech delivery was conducted for 50 (16.6%) and 68 (22.7%) of the first and the second baby respectively. Instrumental vaginal delivery was the mode for 8 (2.7%) of the first baby and 2 (0.7%) of the second baby. Overall, 218 (72.7%) of twins were both delivered vaginally. This is shown in Table 4.

The caesarean section rate for twin delivery was 27.3%. Emergency caesarean section for delivery of both the babies was carried out in 67 (22.3%) of the cases while elective caesarean section for both the babies accounted for 3 (1.0%). Combined vaginal and abdominal delivery occurred in 12 (4.0%) cases. Of these 82 mothers who had caesarean section, 52 (63.4%) were unbooked while the remaining 30 (36) were booked. The unbooked mothers had a 1.7-fold higher chance of caesarean section compared to the booked mothers. Among the 72 mothers who had caesarean section for delivery of both the babies, 23 (31.9%) were nullipara while 13 (18.1%) were primipara; multipara and grand multipara accounted for 28 (38.8%) and 8 (11.2%) respectively.

**Figure.**
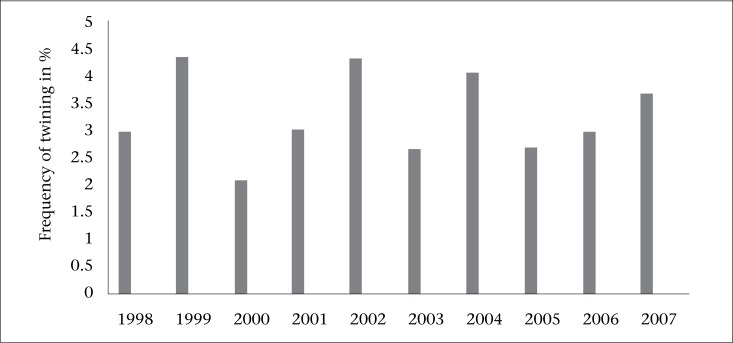
Yearly trend in twin deliveries

**Table 2.** Age and parity of paturients

Age (years)	Number	%	Mean±SD
15-<20	7	2.3	
20-<25	53	17.7	
25-<30	117	39.0	28.4±4.69
30-<35	88	29.3	
35-<40	29	9.7	
40-44	6	2.0	
Total	300	100.0	
Parity	Number	%	Mean±SD
0	77	25.7	
1	57	19.0	
2	61	20.3	2.04±1.84
3	45	15.0	
4	29	9.7	
5	12	4.0	
6	14	4.7	
7	2	0.7	
8	3	1.0	
Total	300	100.0	

There were no statistically-significant associations between the age of the paturients and mode of delivery of the twins (χ^2^=16.695, p=0.337 for the first baby and χ^2^=9.667, p=0.840 for the second baby).

**Table 3.** Obstetric complications associated with twin deliveries

Complication	Number	Percentage
Anaemia	3	1.0
Antepartum haemorrhage	2	0.7
Cord prolapse	9	3.0
Delayed 2^nd^ stage	3	1.0
Hypertensive disorders in pregnancy (Eclampsia, pre-eclampsia, pregnancy-induced hypertension)	28	9.3
Foetal distress	4	1.3
Intrauterine foetal death	2	0.7
Malpositions	24	8.0
Prolonged labour	12	4.0
Premature rupture of membranes	6	2.0
Preterm delivery	119	39.7
None	83	27.6
Others	5	1.7
Total	300	100.0

There was a statistically-significant association between booking status and mode of delivery of both the babies (χ^2^=27.019, p=0.000 for the first baby and χ^2^=24.854, p=0.000 for the second baby). The association between parity and mode of delivery was not statistically significant as shown in Table 5.

The commonest indication for caesarean section was foetal malpresentations occurring in 26 (31.7%) of the caesarean deliveries, followed by hypertensive disorders in pregnancy in 12 (14.7%). Abnormalities in labour (failure to progress and obstructed labour) were the indication in 10 (12.5%)—cord prolapse in 8 (9.8%), previous scar(s) in 7 (8.5%), retained second baby in 6 (7.3%), and foetal distress in 6 (7.3%). Other indications of caesarean section included premature rupture of membranes, antepartum haemorrhage, prevention of HIV, and these accounted for 7 (8.5%) of caesarean sections.

The commonest postpartum complication was primary postpartum haemorrhage which occurred in 15 (5.0%) of mothers. There was one maternal death among mothers with twin gestation during the 10-year period of the review. The cause of death was acute renal failure from antepartum eclampsia.

The mean birthweight of the first baby was 2.40±0.62 kg while the mean birthweight of the second baby was 2.39±0.64 kg. Overall, the mean foetal weight was 2.395±0.63 kg. There was no statistically-significant difference when the weights of the first and the second baby were compared (t=0.343, p=0.732). Of the 600 babies studied, 302 (50.3%) had normal birthweights, 250 (41.7%) were of low birthweights while 30 (5.0%) and 17 (2.8%) were of very low birthweights and extremely low birthweights respectively. Only 1 (0.2%) was macrosomic.

**Table 4.** Modes of delivery

Mode of delivery	Twin			
	1		2	
	Number	%	Number	%
SVD	170	56.7	148	49.3
ABD	50	16.6	68	22.7
C/S	72	24.0	82	27.3
Forceps	6	2.0	1	0.3
Vacuum	2	0.7	1	0.3
Total	300	100.0	300	100.0

ABD=Assisted breech delivery, C/S=Caesarean section, SVD=Spontaneous vaginal delivery

**Table 5.** Associations between variables and mode of delivery of twins

Variable	First baby			Second baby	
	Mean	χ^2^	p value	χ^2^	p value
Age (years)	28.36±4.69	16.695	0.337	9.667	0.840
Parity	2.04±1.84	28.214	0.251	12.882	0.968
Booking status	-	27.019	0.000	24.854	0.000

There were 24 (8.0%) and 37 (12.3%) stillbirths among the first and the second baby respectively. Overall, 61 (10.2%) of the deliveries resulted in stillbirths, giving a stillbirth rate of 102 per 1,000 births. There were statistically-significant associations between booking status of the mothers and Apgar score of the twins in one minute (χ^2^=29.073, p=0.000 for the first baby and χ^2^=23.416, p=0.000 for the second baby).

The female-to-male ratio was 1:1.1 while the mean inter-baby delivery interval was 21.37 minutes. Thirty-six (12.0%) of the second babies were delivered later than 30 minutes of delivery of the first babies.

## DISCUSSION

The overall twining rate in this study of 32.5 per 1,000 deliveries, albeit high, is less than 40.2% reported in a study carried out in four urban hospital settings in South-West Nigeria (5). It is, however, higher than 27.6 reported in Nnewi in the South-East (6), 28 in Jos North-Central area (7), 14.4 in Maiduguri, North-East region (8), and 26 per 1,000 deliveries in Uyo, South-South Nigeria (9). These findings suggest that the rate of twining in Nigeria's federal capital territory is higher than that seen in other regions of the country apart from the Yoruba tribe in South-West Nigeria. The high twining rate in our centre may be due to the mixed population found in Abuja with paturients spread across the different ethnic groups in the country.

Women in the age-group of 25-29 years, with mean age of 28.4±4.69 years, were the majority, accounting for 38.7% of the population studied. This observation is similar to the finding from other studies (3,7). Increased maternal age at conception may be a contributory factor responsible for the observed high incidence in this study since cumulatively 240 (80%) of the paturients were ≥25 years old. Although increased parity is associated with twining rate, low mean parity as found in this study has also been observed among twining mothers (3). Preterm delivery was the commonest obstetric complication observed in the study as was the case in other studies carried out in Jos and Uyo (3,9). It is the most important factor contributing to the increasing perinatal mortality and morbidity in multiple pregnancies (2). The frequency of occurrence of cephalic-cephalic presentations of both babies is similar to findings by Mutihir *et al*. and Nwobodo *et al*. both in Northern Nigeria (3,8).

The overall caesarean section rate in this study is less than findings of 38.9% by Persad *et al*. at a tertiary-care centre in Canada, 43.1% by Mutihir *et al*. in Jos, Nigeria, and 45.0% by Kontopoulos *et al*. in a population-based study in the USA (3,10,11). This was possible because of the high rate of vaginal delivery achieved in our cases due to well-planned delivery for the booked mothers as they were educated on birth preparedness and complication readiness. These were to help them present early in labour, with benefit of adequate intrapartum care and supervised delivery. Caesarean section rate for twin pregnancy is usually higher than that for singletons. One study showed that twin pregnancy has three times higher risk of caesarean section compared to singletons (12). Thus, the caesarean section rate found in the study was within the range reported in other studies (13). Non-vertex presentation of the leading baby was the major factor contributing to the caesarean section rate noticed in the study. Perinatal mortality is said to increase with non-vertex presentations; thus, a liberal approach to caesarean section for breech twin births and particularly for paired breech-breech presentation is strongly advocated by some authorities (14).

This study revealed that almost half of the mothers did not receive antenatal care. Lack of utilization of antenatal care has been associated with poor foetal outcome in twin gestation (15). Antenatal care affords opportunity for assessment of risks as well as planning for the delivery. It is associated with improved outcomes and reduction in perinatal morbidity and mortality (16).

The mean birthweights of the first and the second baby were slightly higher than 2.18 kg and 2.04 kg respectively as reported in another study in South-West Nigeria (17). However, the mean foetal birthweight is comparable to low mean birthweight found in another study (18). The incidence of babies weighing less than 2.5 kg found in this study is comparable to earlier reports (19,20). Thus, more research into measures aimed at preventing preterm deliveries and low birthweight is crucial toward improving the foetal outcome of twin pregnancies in developing countries.

The incidence of combined vaginal-abdominal delivery is similar to findings from other studies (11,19). It has been found to be associated with increased neonatal mortality (10,11). In this study, predisposing factors for combined vaginal-abdominal delivery were mainly unbooked status and non-vertex second baby.

The number of stillbirths was higher in the second baby compared to the first baby. The increased morbidity and mortality associated with the second baby are well-documented (17,18).

The stillbirth rate of 102 per 1,000 births in this study is high, albeit lower than 201 per 1,000 births reported among twin deliveries at Uyo in Nigeria (9). It is outrageously higher than the stillbirth rates found in developed countries, like the United Kingdom and United States of America and also two and a half times higher than the national stillbirth rate of 42 per 1,000 births in Nigeria (21). The findings in this study confirm twin births as a major contributor to perinatal mortality in Nigeria. The high stillbirth rate may have been contributed to by the unbooked status of the women as can be seen from the statistically-significant association between booking status and Apgar score in one minute of the twins.

The female-to-male ratio found in this study is comparable to the finding in Jos, Nigeria (3). The number of retained second babies noted in this study was lower than 16.3% reported in Enugu (22). It is, however, higher than 7.9% in Ife (23). The high incidence noted may be attributable to the large number of unbooked cases in this study.

### Conclusions

Twining rate in delivaries at our centre is high, with a fluctuating trend during the 10 years of the study. Majority of the paturients are of low parity, with preterm delivery as the most common obstetric complication and foetal malpresentations as the leading indication for caesarean section. Successful vaginal delivery of twins is high when the mothers are booked and the presentations of the twins are favourable. The use of antenatal care services and good intrapartum management will help improve outcome in twin pregnancies.
